# Supplementary material for: A Scottish provenance for the Altar Stone of Stonehenge
Source: Nature. 2024 Aug 14;632(8025):570–5. doi: 10.1038/s41586-024-07652-1 (PMC11324516; doi:10.1038/s41586-024-07652-1)
Supplement: Supplementary file 2 — Reporting Summary [file 41586_2024_7652_MOESM2_ESM.pdf]

nature portfolio

Corresponding author(s): Anthony Clarke

Last updated by author(s): May 21, 2024

Reporting Summary

Nature Portfolio wishes to improve the reproducibility of the work that we publish. This form provides structure for consistency and transparency in reporting. For further information on Nature Portfolio policies, see our Editorial Policies and the Editorial Policy Checklist.

Please do not complete any field with "not applicable" or n/a. Refer to the help text for what text to use if an item is not relevant to your study.
For final submission: please carefully check your responses for accuracy; you will not be able to make changes later.

Statistics

For all statistical analyses, confirm that the following items are present in the figure legend, table legend, main text, or Methods section.

n/a	Confirmed	
☐	☒	The exact sample size (n) for each experimental group/condition, given as a discrete number and unit of measurement
☐	☒	A statement on whether measurements were taken from distinct samples or whether the same sample was measured repeatedly
☐	☒	The statistical test(s) used AND whether they are one- or two-sided<br>*Only common tests should be described solely by name; describe more complex techniques in the Methods section.*
☐	☒	A description of all covariates tested
☐	☒	A description of any assumptions or corrections, such as tests of normality and adjustment for multiple comparisons
☐	☒	A full description of the statistical parameters including central tendency (e.g. means) or other basic estimates (e.g. regression coefficient) AND variation (e.g. standard deviation) or associated estimates of uncertainty (e.g. confidence intervals)
☐	☒	For null hypothesis testing, the test statistic (e.g. F, t, r) with confidence intervals, effect sizes, degrees of freedom and P value noted<br>*Give P values as exact values whenever suitable.*
☒	☐	For Bayesian analysis, information on the choice of priors and Markov chain Monte Carlo settings
☒	☐	For hierarchical and complex designs, identification of the appropriate level for tests and full reporting of outcomes
☒	☐	Estimates of effect sizes (e.g. Cohen's d, Pearson's r), indicating how they were calculated

Our web collection on statistics for biologists contains articles on many of the points above.

Software and code

Policy information about availability of computer code

Data collection	Zircon, apatite and rutile U-Pb isotopic and trace element data were collected using an Agilent 8900 triple quadrupole mass spectrometer. Apatite Lu-Hf data was measured using an Agilent 8900 ICP-MS/MS. Further details of analytical set-up can be found in the Methods and Supplementary Information.
Data analysis	For zircon, apatite and rutile U-Pb data reductions Iolite Version 4 (Paton et al 2011) and Isoplot R Version 6.2 (Vermeesch 2018) software were used. For zircon and rutile U-Pb data reduction the "U-Pb Geochronology" Data Reduction Scheme (DRS) was used in Iolite 4. Apatite trace element data was reduced using the "Trace Element" DRS in Iolite 4. Multidimensional scaling plots for zircon age datasets were created using the MATLAB script of Nordsvan et al. (2020), where MATLAB Version 9.11.0 (R2021b Update 2) was used. Apatite U-Pb isotopic data was reduced using the VizualAge UcomPbine DRS of Iolite Version 4. Apatite Lu-Hf ratios were calculate using the LADR software, Version 1.1.7.0 (Build date: 2021-11-03) (Norris & Danyushevsky, 2018) and Isoplot R.

For manuscripts utilizing custom algorithms or software that are central to the research but not yet described in published literature, software must be made available to editors and reviewers. We strongly encourage code deposition in a community repository (e.g. GitHub). See the Nature Portfolio guidelines for submitting code & software for further information.

Data

Policy information about availability of data

All manuscripts must include a data availability statement. This statement should provide the following information, where applicable:

- Accession codes, unique identifiers, or web links for publicly available datasets
- A description of any restrictions on data availability
- For clinical datasets or third party data, please ensure that the statement adheres to our policy

The authors declare that the isotopic and chemical data supporting the findings of this study are available within the paper and its supplementary information files (1-3).

Research involving human participants, their data, or biological material

Policy information about studies with human participants or human data. See also policy information about sex, gender (identity/presentation), and sexual orientation and race, ethnicity and racism.

Reporting on sex and gender	Not applicable
Reporting on race, ethnicity, or other socially relevant groupings	Not applicable
Population characteristics	Not applicable
Recruitment	Not applicable
Ethics oversight	Not applicable

Note that full information on the approval of the study protocol must also be provided in the manuscript.

Field-specific reporting

Please select the one below that is the best fit for your research. If you are not sure, read the appropriate sections before making your selection.

☒ Life sciences ☐ Behavioural & social sciences ☐ Ecological, evolutionary & environmental sciences

For a reference copy of the document with all sections, see nature.com/documents/nr-reporting-summary-flat.pdf

Life sciences study design

All studies must disclose on these points even when the disclosure is negative.

Sample size	Sample size for zircon, apatite and rutile data was dictated by the availability of sufficiently sized grains for laser-ablation within the thin-sections analysed. For compilations of zircon U–Pb ages from source terranes we used compilations by Fairey et al. (2018) and Stevens and Baykal (2021). For zircon U–Pb ages from Old Red Sandstone basins we calculated concordia ages based on reported isotopic data and used the concordia log distance % approach (≤ 10 % discordant) to filter ages.
Data exclusions	No data was excluded from the study.
Replication	All reported ages for secondary reference material are within two standard error of accepted values. Furthermore, we detail our analytical and instrument set-up within the methods.
Randomization	Randomization was not applicable to this study. We collected isotopic and chemical data from thin-sections of rock to determine the provenance of the Altar Stone - negating the need for randomization
Blinding	Blinding was not applicable to this study as we collected isotopic and chemical data for thin-sections of rock.

Reporting for specific materials, systems and methods

We require information from authors about some types of materials, experimental systems and methods used in many studies. Here, indicate whether each material, system or method listed is relevant to your study. If you are not sure if a list item applies to your research, read the appropriate section before selecting a response.

Materials & experimental systems

n/a	Involved in the study
☒	☐ Antibodies
☒	☐ Eukaryotic cell lines
☐	☒ Palaeontology and archaeology
☒	☐ Animals and other organisms
☒	☐ Clinical data
☒	☐ Dual use research of concern
☒	☐ Plants

Methods

n/a	Involved in the study
☒	☐ ChIP-seq
☒	☐ Flow cytometry
☒	☐ MRI-based neuroimaging

Palaeontology and Archaeology

Specimen provenance

This work analysed two 30 µm polished thin sections of the Altar Stone, MS3 and 2010K.240. Both thin sections were prepared from rock fragments collected from Stonehenge, U.K. during archaeological digs of the Altar Stone during the 20th century and were loaned and sampled with permission of National Museum of Wales and Salisbury Museum. We also analysed two sections of Old Red Sandstone rock from the Orcadian Basin (CQ1 and AQ1). CQ1 is from Cruaday, Orkney (59°04'34.2" N, 3°18'54.6" W), and AQ1 is from near Spittal, Caithness (58°28'13.8" N, 3°27'33.6" W). Both Orcadian Basin geological rock samples were purchased from the UK company: Natural Wonders ltd Registered office 20 Grape Lane, Whitby, YO22 4BA, North Yorkshire, Company Registration Number 05427798.

Specimen deposition

MS3 and 2010K.240. were received on loan from National Museum of Wales and Salisbury Museum respectively.

Dating methods

New dates are provided. We report in-situ zircon, apatite and rutile U-Pb ages (calculated as single-spot concordia ages) from MS3 and 2010K.240. From 2010K.240 we report apatite Lu-Hf ages. For Orcadian Basin samples we report apatite U-Pb ages.

☒ Tick this box to confirm that the raw and calibrated dates are available in the paper or in Supplementary Information.

Ethics oversight

Thin sections MS3 and 2010K.240 were analysed with permission of the National Museum of Wales and Salisbury Museum respectively.

Note that full information on the approval of the study protocol must also be provided in the manuscript.

Plants

Seed stocks

Not applicable

Novel plant genotypes

Not applicable

Authentication

Not applicable

This checklist template is licensed under a Creative Commons Attribution 4.0 International License, which permits use, sharing, adaptation, distribution and reproduction in any medium or format, as long as you give appropriate credit to the original author(s) and the source, provide a link to the Creative Commons license, and indicate if changes were made. The images or other third party material in this article are included in the article's Creative Commons license, unless indicated otherwise in a credit line to the material. If material is not included in the article's Creative Commons license and your intended use is not permitted by statutory regulation or exceeds the permitted use, you will need to obtain permission directly from the copyright holder. To view a copy of this license, visit http://creativecommons.org/licenses/by/4.0/
