# Supplementary material for: A Scottish provenance for the Altar Stone of Stonehenge
Source: Nature. 2024 Aug 14;632(8025):570–5. doi: 10.1038/s41586-024-07652-1 (PMC11324516; doi:10.1038/s41586-024-07652-1)
Supplement: Supplementary file 6 — Supplementary Information 4: Summary of analyses. Summary table of analyses undertaken in this work on samples from the Altar Stone and the Orcadian Basin. Supplementary Information 5: Summary of zircon U–Pb reference material. A summary table of analyses was obtained for zircon U–Pb secondary reference material run during this work. Supplementary Information 6: Kolmogorov–Smirnov test results. Table of D and P values for the Kolmogorov–Smirnov test on zircon ages from the Altar Stone and potential source regions. Supplementary Information 7: Kolmogorov–Smirnov test results, with Monte Carlo resampling. Table of D and P values for the Kolmogorov–Smirnov test (with Monte Carlo resampling) on zircon ages from the Altar Stone and potential source regions. Supplementary Information 8: Summary of apatite U–Pb reference material. A summary table of analyses was obtained for the apatite U–Pb secondary reference material run during this work. [file 41586_2024_7652_MOESM6_ESM.pdf]

Supplementary Information 4

A summary of analyses performed on each Altar Stone and Orcadian Basin sample.

X denotes the analysis undertaken.

Altar Stone Samples

Sample	Automated Mineralogy	Zircon U–Pb	Rutile U–Pb	Apatite U–Pb	Apatite Lu–Hf	Apatite Trace Elements
MS3	X	X	X	X		X
2010K.240	X	X			X	

Orcadian Basin Samples

Sample	Automated Mineralogy	Zircon U–Pb	Rutile U–Pb	Apatite U–Pb	Apatite Lu–Hf	Apatite Trace Elements
Spittal,Caithness (AQ1)	X			X		
Cruaday, Orkney (CQ1)	X			X		

Supplementary Information 5 . Summary of Zircon U–Pb Reference Material

Session 1 (MS3)

Reference	Primary Reference	Weighted Mean Age (Ma)*	± 2σ	MSWD*	*P**	*n*
**91500**	GJ1	**1059**	**10**	**0.72**	**0.69**	**10**
Published age		1063.78	0.65	*Wiedenbeck et al., 1995*		
**Plešovice**	GJ1	**336**	**3**	**0.69**	**0.78**	**15**
Published age		337.13	0.37	*Slama et al., 2008*		
**OG1**	GJ1	**3468**	**4**	**0.73**	**0.75**	**16**
Published age		3465.40	0.60	*Stern et al., 2009*		
**Maniitsoq**	GJ1	**3014**	**6**	**0.65**	**0.85**	**17**
Published age		3008.70	0.60	*Marsh et al., 2019*		

Session 2 (2010K.240)

Reference	Primary Reference	Weighted Mean Age (Ma)*	± 2σ	MSWD*	*P**	*n*
**91500**	GJ1	**1060**	**3**	**1.20**	**0.23**	**24**
Published age		1063.78	0.65	*Wiedenbeck et al., 1995*		
**Plešovice**	GJ1	**338**	**1**	**1.20**	**0.27**	**18**
Published age		337.13	0.37	*Slama et al., 2008*		
**OG1**	GJ1	**3466**	**7**	**0.78**	**0.79**	**29**
Published age		3465.40	0.60	*Stern et al., 2009*		

*Where MSWD and P are the mean square of the weighted deviation value and the associated MSWD age homogeneity test chi-squared P-value, both metrics are one-sided measurements.

Supplementary Information 6. KS Test Results. This KS test is two-sided (i.e. this test checks for any difference in distribution). The bottom half shows P-values (Highlighted green values are *P*>0.05). The top table half shows the D-values (Highlighted in blue are the associated D-values for *P*>0.05).

	Altar Stone	East Avalonia	Laurentia	Ganderia	Megumia	West Avalonia	Armorica-Cadomia	Orcadian Basin	Anglo-Welsh Basin	Dingle Peninsula Basin	Midland Valley	Svalbard
**Altar Stone**		0.51	0.16	0.40	0.68	0.61	0.57	0.10	0.45	0.43	0.32	0.13
**East Avalonia**	0.00		0.53	0.12	0.21	0.15	0.18	0.50	0.28	0.21	0.33	0.45
**Laurentia**	0.10	0.00		0.42	0.71	0.63	0.60	0.23	0.47	0.45	0.41	0.21
**Ganderia**	0.00	0.00	0.00		0.29	0.24	0.18	0.39	0.29	0.27	0.25	0.34
**Megumia**	0.00	0.00	0.00	0.00		0.23	0.15	0.68	0.30	0.28	0.50	0.63
**West Avalonia**	0.00	0.00	0.00	0.00	0.00		0.22	0.61	0.25	0.21	0.43	0.55
**Armorica-Cadomia**	0.00	0.00	0.00	0.00	0.00	0.00		0.57	0.26	0.25	0.39	0.52
**Orcadian Basin**	0.72	0.00	0.00	0.00	0.00	0.00	0.00		0.44	0.42	0.37	0.11
**Anglo-Welsh Basin**	0.00	0.00	0.00	0.00	0.00	0.00	0.00	0.00		0.10	0.26	0.35
**Dingle Peninsula Basin**	0.00	0.00	0.00	0.00	0.00	0.00	0.00	0.00	0.33		0.25	0.37
**Midland Valley**	0.00	0.00	0.00	0.00	0.00	0.00	0.00	0.00	0.00	0.00		0.34
**Svalbard**	0.36	0.00	0.00	0.00	0.00	0.00	0.00	0.06	0.00	0.00	0.00	

Supplementary Information 7 . KS Test Results, with Monte-Carlo resampling. This KS test is two-sided (i.e. this test checks for any difference in distribution). The bottom half of the table shows P-values (Highlighted green values are *P*>0.05). The top half of the table shows two standard deviation values on the KS test P-value (highlighted blue values are two standard deviation values for those *P*>0.05).

	**Altar Stone**	**East Avalonia**	**Laurentia**	**Ganderia**	**Megumia**	**West Avalonia**	**Armorica-Cadomia**	**Orcadian Basin**	**Anglo-Welsh Basin**	**Dingle Peninsula Basin**	**Midland Valley**	**Svalbard**
**Altar Stone**		0.00	0.05	0.00	0.00	0.00	0.00	0.16	0.00	0.00	0.00	0.10
**East Avalonia**	0.00		0.00	0.00	0.00	0.00	0.00	0.00	0.00	0.00	0.00	0.00
**Laurentia**	0.11	0.00		0.00	0.00	0.00	0.00	0.00	0.00	0.00	0.00	0.00
**Ganderia**	0.00	0.00	0.00		0.00	0.00	0.00	0.00	0.00	0.00	0.00	0.00
**Megumia**	0.00	0.00	0.00	0.00		0.00	0.00	0.00	0.00	0.00	0.00	0.00
**West Avalonia**	0.00	0.00	0.00	0.00	0.00		0.00	0.00	0.00	0.00	0.00	0.00
**Armorica-Cadomia**	0.00	0.00	0.00	0.00	0.00	0.00		0.00	0.00	0.00	0.00	0.00
**Orcadian Basin**	0.67	0.00	0.00	0.00	0.00	0.00	0.00		0.00	0.00	0.00	0.03
**Anglo-Welsh Basin**	0.00	0.00	0.00	0.00	0.00	0.00	0.00	0.00		0.19	0.00	0.00
**Dingle Peninsula Basin**	0.00	0.00	0.00	0.00	0.00	0.00	0.00	0.00	0.38		0.00	0.00
**Midland Valley**	0.00	0.00	0.00	0.00	0.00	0.00	0.00	0.00	0.00	0.00		0.00
**Svalbard**	0.33	0.00	0.00	0.00	0.00	0.00	0.00	0.06	0.00	0.00	0.00	

Supplementary Information 8. Summary of Apatite U–Pb Reference Material

Session 1 (MS3)

Reference	Primary Reference	Age (Ma)	± 2σ	MSWD*	*P**	*n*
**Otter Lake**	**MAD-1**	**926**	**7**	**1.7**	**0.06**	**15**
Published age		913	7	*Barfod et al., 2005*		
**FC-1**	**MAD-1**	**1109**	**16**	**1.5**	**0.08**	**17**
Published age		1099.1	0.6	*Schmitz et al., 2003*		
**Mount McClure**	**MAD-1**	**526**	**8**	**1.5**	**0.10**	**17**
Published age		523.5	1.5	*Schoene at al., 2006; Thompson et al., 2012*		

Session 2 (Orcadian Basin Samples CQ1 and AQ1)

Reference	Primary Reference	Age (Ma)	± 2σ	MSWD*	*P**	*n*
**Durango**	**MAD-1**	**28**	**3**	**0.55**	**0.90**	**15**
Published age		31.44	0.18	*McDowell et al., 2005*		
**FC-1**	**MAD-1**	**1088**	**12**	**1.4**	**0.13**	**17**
Published age		1099.1	0.6	*Schmitz et al., 2003*		
**Mount McClure**	**MAD-1**	**527**	**5**	**0.6**	**0.86**	**16**
Published age		523.5	1.5	*Schoene et al., 2006; Thompson et al., 2012*		

*Where MSWD and P are the mean square of the weighted deviation value and the associated MSWD age homogeneity test chi-squared P-value, both metrics are one-sided.
